# Vacuolar Myopathy Associated to CACNA1S Mutation as a Rare Cause of Late-Onset Limb-Girdle Myopathy: A Case Report

**DOI:** 10.7759/cureus.18873

**Published:** 2021-10-18

**Authors:** Juan Carlos López-Hernández, Javier A Galnares-Olalde, Edmar Benitez-Alonso, Raul E Alcalá, Edwin Steven Vargas-Cañas

**Affiliations:** 1 Neuromuscular Diseases, Instituto Nacional de Neurología y Neurocirugía Manuel Velasco Suárez, Mexico City, MEX; 2 Neurology, Instituto Nacional de Neurología y Neurocirugía Manuel Velasco Suárez, Mexico City, MEX; 3 Neurogenetics Department, Instituto Nacional de Neurología y Neurocirugía, Mexico City, MEX; 4 Neuromuscular Disease Department, Instituto Nacional de Neurología y Neurocirugía, Mexico City, MEX

**Keywords:** primary periodic paralysis, vacuolar myopathy, limb-girdle myopathy, gene, cacna1s

## Abstract

Late-onset limb-girdle myopathies pose a diagnostic challenge. The most common etiologies are inflammatory, followed by genetic and metabolic. Rare cases include limb-girdle dystrophies and permanent myopathies (vacuolar), such as those associated with hypokalemic periodic paralysis (HypoPP). We present the case of a 59-year-old male who initiated with episodic acute severe weakness when he was 11, during which serum potassium levels of <2.5 meq/L were revealed during workup. Potassium reposition reversed these episodes. They occurred every three to five years, and the last episode was five years prior to the current illness. When he was 58, he presented progressive pelvic girdle weakness. On examination, he presented decreased strength in the iliopsoas and quadriceps. The laboratory results showed mildly elevated creatine kinase. Muscle biopsy revealed a vacuolar myopathy, and genetic testing identified a pathogenic variant in the CACNA1S gene, locus 1q32.1 *[c.3716G> A (p.Arg1239His), heterozygous state].*

## Introduction

Late-onset limb-girdle myopathies commonly pose a diagnostic challenge. First, there is no clear definition of “late onset,” and most papers use the age of 40 years as the cutoff value. Second, differential diagnosis is wide, including hereditary disorders, such as oculopharyngeal, Becker, limb-girdle, facioscapulohumeral and myotonic dystrophies, and Pompe disease; myofibrillar and mitochondrial myopathies; and acquired causes, such as inflammatory myopathies, nonspecific myositis, and slow late-onset nemaline myopathy [1].

Permanent vacuolar myopathy secondary to hypokalemic periodic paralysis (HypoPP) is a rare cause of late-onset girdle myopathy, and few cases have been reported in the literature. Definitive diagnosis is supported by muscle biopsy and molecular genetic analysis [2].

HypoPP is an autosomal dominantly inherited channelopathy with a prevalence of 1:100,000, being more frequent in men than in women [2]. It is caused by mutations in the α-subunit of the calcium channel Cav1.1 and the sodium channel Nav1.4, which are encoded by the genes CACNA1S and SCN4A, respectively. The most common mutations are p.R528H and p.R1239H in the CACNA1S gene (type 1), which together account for 70%-80% of cases, whereas 10% of HypoPP carriers have SCN4A mutations (type 2). The remaining cases are associated with point mutations [3,4].

The characteristic clinical features of HypoPP are acute flaccid weakness episodes associated with low serum potassium levels (<2.5 mEq/L). These can either occur spontaneously or be caused by physical activity or high carbohydrate meals. The duration of symptoms ranges from minutes to days, and episodes may happen once in a lifetime or present several daily episodes [4,5]. Classically, patients with HypoPP have normal muscle strength between episodes, although few patients can develop permanent muscle weakness after several years having continuous episodes. Patients with HypoPP rarely present myopathy in advanced ages. In those cases, weakness affects pelvic limb-girdle muscles, despite previous periodic paralysis attacks. Options for preventative therapy include dietary/lifestyle modifications (e.g., avoidance of potassium-rich foods, cold exposure, strenuous exercise, and fasting) and the carbonic anhydrase inhibitors dichlorphenamide and acetazolamide. The frequency of limb-girdle myopathy in patients with HypoPP is unknown [6].

We present the case of a patient with periodic paralysis with late-onset limb-girdle myopathy at an advanced age.

## Case presentation

We present the case of a 59-year-old male without a relevant family history. When he was 11, he had an acute flaccid weakness episode without cranial nerve involvement after intense physical activity and high carbohydrate consumption. He was admitted to his local emergency department where workup revealed low serum potassium levels (<2.5 mEq/L), and weakness improved after potassium reposition. Similar episodes occurred every three to five years, also occurring after intense physical activity or high carbohydrate consumption. Despite extensive workup during adolescence, most metabolic and endocrine causes were excluded. Management included avoiding strenuous exercise and low carbohydrate consumption, as well as potassium salt tablets. The patient was episode-free for more than 20 years. When he turned 58, the patient noticed progressive weakness that noted climbing stairs and getting up from chairs.

**Figure 1:**
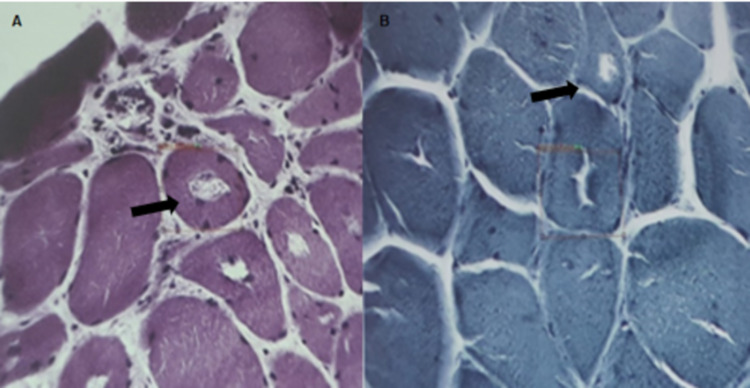
Deltoid muscle biopsy. A. Hematoxylin and eosin staining with chronic myopathic changes and multiple vacuoles (arrow). B. Gomori's modified trichrome stain with multiple vacuoles (arrow).

Six months after this, the patient was unable to get up from the toilet. He was evaluated in our clinic, where neurologic examination demonstrated pelvic girdle muscle atrophy, without compromising facial, cervical, and upper limb muscles. Strength evaluation demonstrated weakness in the iliopsoas, quadriceps, hip adductors, hip abductors, and anterior tibialis (Medical Research Council (MRC) score: 2/5). Reflex and sensory examinations were unremarkable. Laboratory tests revealed glycosylated hemoglobin of 6.4% and creatine kinase of 650 U/L. Left deltoid muscle biopsy reported conserved fascicles, increased interfascicular connective tissue, myopathic changes, and abundant fibers with vacuoles, without observing peripheral or perivascular inflammatory reaction (Figure 1). 

Molecular analysis by sequencing and multiplex ligation-dependent probe amplification (MLPA) of 201 genes demonstrated one pathogenic variant in the CACNA1S gene, locus 1q32.1 [c.3716G>A (p.Arg1239His), heterozygous status].

## Discussion

**Figure 2:**
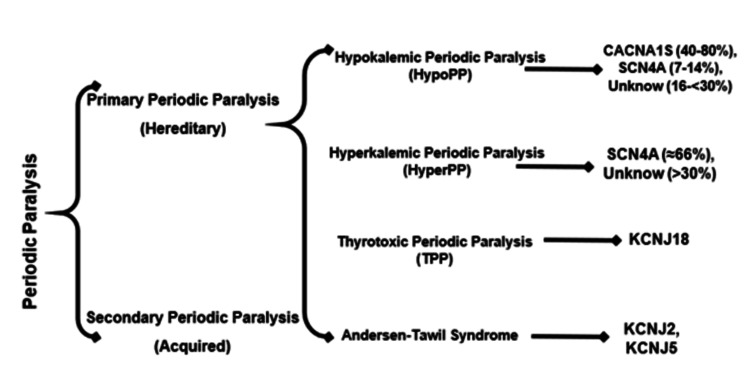
Classification of the etiological causes of periodic paralysis.

Periodic paralysis comprises a rare group of neuromuscular disorders classified as channelopathies that affect the skeletal muscle. The causes are divided into acquired (such as toxic and metabolic) and hereditary. Hereditary causes, also known as primary periodic paralysis, are further divided into hyperkalemic periodic paralysis (HyperPP) and hypokalemic periodic paralysis (HypoPP). The latter is characterized by triad periodic paralysis, ventricle arrhythmias, and developmental abnormalities (Figure 2) [4,5].

HypoPP is an infrequent disease, with an incidence of 1 case per 100,000 persons/year, with an autosomal dominant inheritance. The most common mutations are p.R528H and p.R1239H in the CACNA1S gene (type 1), which together account for 70%-80% of cases. Of the HypoPP carriers, 10% have SCN4A mutations (type 2), and the remaining cases are associated with unknown point mutations [4,5]. In our case, genetic analysis revealed a mutation in the CACNA1S, which has not been reported previously.

Four clinical phenotypes related to CACNA1S mutation HypoPP have been described. The most common phenotype (56%), also known as classical, presents with acute periodic paralysis episodes with complete recovery between attacks. The second phenotype includes acute periodic paralysis episodes with permanent weakness, occurring in 31%. The other two phenotypes include permanent weakness without acute episodes (7%) and asymptomatic patients (5%) [6,7].

HypoPP attacks generally start during adolescence and include acute symmetric weakness of the four limbs, even reaching quadriplegia, which evolves in hours or days without respiratory, pharyngeal, or cranial nerve involvement. The clinical picture resembles Guillain-Barré syndrome with a faster and more severe course. Attacks are triggered by many causes, including exercise, stress, high intake of carbohydrate meals, and thyroid abnormalities, and laboratory workup reveals serum potassium levels less than 2.5 mEq/L. Most patients recover gradually in hours or days after potassium reposition, although some patients may not recover completely as mentioned earlier [4-6].

Our case is of special interest, because our patient had few attacks in his entire life and between each attack, he fully recovered muscle strength; the last attack prior to his current condition was five years ago. The patient presents pelvic girdle weakness, with great involvement of the iliopsoas and quadriceps muscles rather than upper limb-girdle weakness, with trophic changes and slightly elevated CK levels. In our differential diagnosis, an inflammatory myopathy was considered, meeting probable criteria by the ACR/EULAR criteria considering only clinical variables [7]. Moreover, inflammatory myopathies are more common than late-onset genetic myopathies. The reported incidence of inflammatory myopathy is 2.18-7.7 cases per 100,000 inhabitants and increases with age, reporting nine cases per 100,000 inhabitants [8,9].

A muscle biopsy was performed with the intention of finding a characteristic inflammatory reaction, but we observed vacuolar myopathy without an inflammatory reaction. Vacuolar myopathy is not pathognomonic for HypoPP, but it can be observed in patients with late-onset myopathy, including some genes related to limb-girdle dystrophies [2]. In the era of NGS studies, a study reported not only the genes associated with vacuolar myopathy, namely, GNE, LDB3/ZASP, MYOT, DES, and GAA, but also the genes that are not regularly associated with severely altered autophagy, namely, FKRP, DYSF, CAV3, COL6A2, GYG1, and TRIM32 [10].

Vacuolar myopathy in patients with HypoPP is thought to be a marker of myopathy, although vacuoles are seen in patients without permanent muscle weakness. It seems to affect mostly pelvic girdle muscles. Pathological studies suggest that the vacuoles arise from the sarcoplasmic reticulum and the tubular system as the result of the proliferation and degeneration of these membranous organelles. Other morphological changes may also be observed, such as tubular aggregates that originated under the muscle ﬁber membrane.

## Conclusions

HypoPP is a rare genetic dominant disorder. This patient demonstrates that vacuolar myopathy should be considered as a differential diagnosis in late-onset limb-girdle myopathy workup with a history of HypoPP. Future research should consider new treatments for this pathology.
